# Breastfeeding, Walking Onset, and Abdominal Obesity Are Determinants of Physical Fitness among Latin American and Spanish Schoolchildren: A Cross-Cultural Study

**DOI:** 10.3390/epidemiologia5030022

**Published:** 2024-06-26

**Authors:** Karina E. Andrade-Lara, José Carlos Cabrera Linares, Juan Antonio Párraga Montilla, Alexander Mayanquer-Lara, Manuel Lucena Zurita, Pedro Ángel Latorre Román

**Affiliations:** 1 Department of Musical, Plastic and Corporal Expression, University of Jaén, 23071 Jaén, Spain; karinandrade9011@gmail.com (K.E.A.-L.); jccabrer@ujaen.es (J.C.C.L.); platorre@ujaen.es (P.Á.L.R.); 2 Unidad Educativa Víctor Manuel Guzmán, Ibarra 100104, Ecuador; aalexandryman8825@hotmail.com; 3 Escuelas Profesionales de la Sagrada Familia, 23400 Úbeda, Spain; mlucena@fundacionsafa.es

**Keywords:** fitness level, school-aged children, children obesity, nursing, gait initiation

## Abstract

Objective: To comp+are levels of physical fitness between Ecuador and Spain and identify whether breastfeeding period, walking onset, and abdominal obesity are determinants of physical performance in schoolchildren from Ecuador and Spain. Methods: a total of 352 schoolchildren (6–12 years old) from Ecuador (n = 176) and Spain (n = 176) joined in this study. Anthropometric measures, socio-demographic characteristics, and physical fitness were evaluated. Results: Spanish schoolchildren showed better performance in handgrip strength, standing long jump, and 25 m sprint (*p* = 0.021; *p* < 0.001; *p* < 0.001; *p* < 0.001, respectively). Furthermore, Spanish children showed better cardiorespiratory fitness (*p* < 0.001) and a higher ${VO}_{2}$ max (*p* = 0.002) with regards to their peers. In addition, children from Ecuador and Spain showed an influence of breastfeeding period (*p* ranged from <0.001 to 0.043) and walking onset (*p* ranged from <0.001 to 0.032) on physical performance. Moreover, physical fitness components were protective factors of abdominal obesity in Ecuadorian and Spanish schoolchildren (*p* ranged from =0.001 to 0.049). Conclusions: Our findings revealed the influence of the infancy period and the onset of walking on children’s physical performance, highlighting the importance of these factors in motor development during early childhood and also their influence in middle childhood and throughout adulthood.

## 1. Introduction

In recent years, physical fitness (PF) has been considered a biomarker (biological marker), since it assesses the impact of exercise on systems, tissues, and organs, as well as indicates the body’s capacity to respond to different levels of physical activity, training loads, or performance in daily physical activities [1]. Specifically, scientific evidence concludes that PF serves as a strong indicator of health in children and adolescents [2,3].

In this context, PF is a cornerstone of children’s development, playing a pivotal role in promoting overall health and well-being [4]. Early childhood is a crucial time of rapid growth and development of motor and cognitive skills, especially gross and fine motor skills, which are the first building blocks for the development of children’s PF and performance in various aspects of their lives. Thus, the stimulation of better long-term PF seems to be related to early childhood factors such as breastfeeding time [5] and walking onset [6].

Regarding the first years of life, the onset of walking is commonly characterized by instability, bowed legs, and the use of outstretched arms to keep balance, together with a rapid and uneven sequence of steps with flat feet [7]. At this stage, toddlers undergo gradual and dynamic changes, including rapid growth and development of anatomical, neuromuscular, and sensory systems [8]. Thus, the onset of walking represents a crucial step in a child’s motor development, with powerful connections to their social and perceptual domains [9].

Concerning walking onset, reports indicate that this developmental milestone typically occurs around the age of 12 months. However, it is noteworthy that approximately 10% of toddlers with normal development may not achieve independent walking until the age of 14.4 months or later [10]. Previous research demonstrated an association with a small effect between early onset of walking and improved motor development in healthy children [6,11]. Kuh et al. found, in a prospective study involving a sample of 1374 British men and 1410 British women, that height and weight measures assessed when children started walking had an influence on cognitive functioning and motor coordination, demonstrating that growth and development in early childhood affect individual performance in middle childhood, underlining the natural diversity of children’s developmental processes [12].

On the other hand, early nutritional factors, such as breastfeeding, have beneficial effects on cardiovascular risk factors such as adiposity [13] and blood pressure [14]. Conversely, undernutrition during infancy and childhood is correlated with lower cardiorespiratory fitness in adulthood [15,16,17]. Tambalis et al. [5] highlighted that exclusive breastfeeding (breast milk) ≥ 6 months showed a positive influence on PF performance during infancy. Labayen et al. [18] suggested that longer breastfeeding may have an influence on cardiorespiratory fitness in children and adolescents in later stages of life.

Thus, childhood is the period during which healthy and unhealthy physical activity habits are established [19]. However, the increasing prevalence of physical inactivity among children has become a worrying trend, contributing significantly to the rise in childhood obesity [20,21]. In this context, the waist-to-height ratio (WtHR) has been identified as a pediatric indicator of health [22]. This metric holds significant relevance as it is strongly correlated with an elevated risk of cardiovascular diseases among children [23]. Moreover, WtHR is an indicator of abdominal obesity (AO) [24] that is considered a superior health indicator due to its heightened sensitivity as an early warning signal. Thus, AO might be a reliable indicator of cardiovascular disease risk in children [25]. 

The increasing prevalence of childhood obesity over the past 50 years has reflected dramatic socio-cultural changes, such as increasing urbanization, economic growth, modernization of food markets, and globalization [26]. Culture plays a key role in determining the somatotypes, diets, and habitual physical activity practices of adults and children in all societies [27]. However, in today’s globalized society, the prevalence of unhealthy habits is shared by different cultures around the world [28]. In this context, cross-cultural research is emerging as a tool of scientific interest to understand how diverse and culturally different environments can affect the healthy habits embedded in the lifestyles of each cultural context [23,29]. Thus, factors such as obesity and overweight are of great importance to the scientific community and to the economic development of governments in each cultural context [30]. Currently, there is limited information on AO and postnatal factors in children and their association with PF through cross-cultural analysis. Information is particularly scarce when comparing Latin American and European contexts.

In this regard, the relationship between the fitness levels of European and Latin American countries is strongly linked to their respective Human Development Index (HDI) (indicators such as health, education, and income) [31]. A higher HDI often correlates with superior health conditions and, by extension, a more physically active population [32]. European countries, such as Spain, have a very high HDI (0.904) and a gross domestic product of approximately USD 1.4 trillion [31], and they tend to show better levels of PD, which is influenced by widespread availability of sports facilities, health policies, and a stronger social emphasis on physical activity, leading to more active participation in maintaining a healthy lifestyle [33]. In contrast, many Latin American countries, such as Ecuador, despite having a high HDI (0.740) and a gross domestic product (nominal) of approximately $105 billion USD [31], face disparities in access to health care and sporting resources, leading to significant variations in the overall fitness levels of their populations [34]. Thus, less economically developed countries have a lower prevalence of physical activity, while more economically developed countries have a higher prevalence of physical activity [35]. 

These gaps underline the need for targeted efforts to promote fitness in regions where levels may be comparatively lower. Furthermore, this research seeks to contribute valuable insights into the complex relationship between early-life factors, AO, and PF performance in schoolchildren. Additionally, these finding may have implications for public health interventions, policy development, and the promotion of healthier lifestyles in diverse socio-cultural settings. 

Therefore, the aim of this study was to compare levels of PF between Ecuador and Spain and identify whether breastfeeding period, walking onset, and AO are determinants of physical performance in schoolchildren from Ecuador and Spain. We hypothesized that (a) Spanish schoolchildren will show a better physical condition than Ecuadorian schoolchildren, and (b) breastfeeding period, walking onset, and AO will not show an association with physical performance.

## 2. Materials and Methods

### 2.1. Participants

This cross-sectional study included 352 schoolchildren (6–12 years old; age = 8.54 ± 1.79 years old; 51.4% boys) from Ecuador (n = 176) and Spain (n = 176). The participants were selected for convenience from four schools (two per country) of different urban and rural areas. Parents and teachers were informed about the study and signed an informed consent form to allow their children to join in this research. The inclusion criteria for participating in the study were (a) signed informed consent from the parents and (b) no physical impairments (e.g., chronic pain disorders, sensory impairments, mobility limitations or asthma), and/or intellectual disabilities (e.g., Down’s syndrome or autism spectrum disorders). This study was approved by the Ethics Committee of the University of Jaén (reference code: JUN.21/7.TES), and the ethical recommendations approved by the Declaration of Helsinki were followed. 

### 2.2. Materials and Procedures

Socio-demographic information was provided by the parents using an ad hoc questionnaire. It consisted of questions about walking onset and the breastfeeding period, as well as socio-demographic questions such as socio-economic status, level of education, marital status, if the parents practiced any sport, etc.

#### 2.2.1. Anthropometric Measures

Body weight (kg) was recorded using an electronic scale (OMRON BF 51) and height (m) was measured with a stadiometer (Seca 222, Hamburg, Germany). Body mass index (BMI) was calculated by dividing the body mass (kg) by the square of the body height (m). Waist circumference (WC) was measured following the guidelines of the International Society for Advances in Kineanthropometry [36]. In addition, the waist-to-height ratio (WtHR) was calculated (WC/height) and a cutoff point ≥ 0.50 was considered as AO [23,37].

#### 2.2.2. Physical Fitness

The PF test selected in the current study was used in previous studies [2,38]. These tests focused on assessment of PF such as strength, endurance, and speed.

Handgrip strength was assessed using a manual dynamometer with adjustable grip (T.K.K. 5101 Grip-D; Takey, Tokyo, Japan) (kg) and the protocol of Ruiz et al. [39] for hand strength assessment was followed. Each participant completed two trials with their right and left hands. The score was obtained from the average of the dynamometry values of both hands. 

A standing long jump was performed to evaluate the explosive strength of the lower limbs [40]. Moreover, a 25 m sprint test was performed on a flat surface. To register the time (s), two photocells (WITTY, Microgate Srl, Bolzano, Italy) were used and located in the beginning and the end of the established 25 m track. Each participant performed two trials in each test. The best trial was recorded.

Aerobic capacity was evaluated using the Léger test [41]. The test is structured between two 20 m lines, where participants run forward and backward according to a beep that indicates an increase in speed. The test ends when the participant arrives on the line after the beep and the period at which they stopped is recorded. Maximum oxygen (VO_2_ max) was obtained using the following equation: VO_2_ max = (31.025) + (3.238 × X) − (3.248 × A) + (0.1536 × A × X), where X is the speed that corresponds to the period in which the participant stopped and A is the age of the participant [41]. Finally, the perception of effort was measured using Borg’s [42] subjective perception of effort scale at the end of the Léger test. The scale score ranges from 0 to 10, where 0 means lowest effort and 10 is the highest effort in the test.

### 2.3. Procedure

Firstly, the parents signed an informed consent to allow their children to join in the study. Secondly, the children gave consent to the research team before starting the assessment sessions. The evaluation protocol was carried out in two sessions in the school. Before starting the testing sessions, the children performed a warm-up session led by researchers for 10 min. In the first session, the anthropometric measures, handgrip strength test, and 25 m sprint were carried out. During the second session, the Léger test and the standing long jump were evaluated. Students carried out preliminary trials for each test, and the research team also demonstrated how to perform each test properly before starting the assessment. Children were motivated and encouraged verbally to perform at their best. 

### 2.4. Statistical Analysis

The data were analyzed using SPSS Statistics v.24.0 for Windows (Chicago, IL, USA). The results are expressed as means, standard deviation (SD), and percentages. Normal distribution and homogeneity tests (Kolmogorov–Smirnov test and Levene’s test, respectively) were performed on all data prior to analysis. Differences between groups were assessed using analysis of variance (ANOVA). The association of breastfeeding period, walking onset, and AO with physical performance was evaluated across the β coefficient using the regression logistic model and partial correlation analysis (adjusted by sex and age). In addition, a Z score was calculated for the average PF obtained from the average of all fitness tests transformed into a Z score. The logistic regression was used between dichotomic variables from the AO score (without <0.50/with obesity > 0.50). The significance level was set at *p* < 0.05 and the confidence interval at 95.

## 3. Results

**Figure 1:**
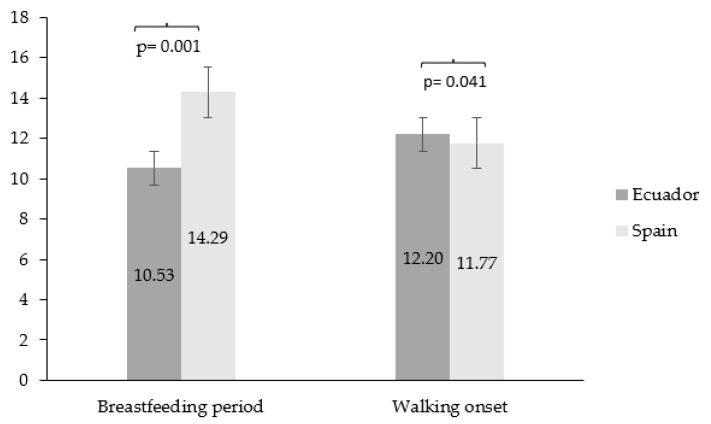
Comparison of breastfeeding period and walking onset between countries.

Figure 1 shows socio-demographic characteristics that indicate that the Spanish children had a longer breastfeeding period than the Ecuadorian children (*p* = 0.001). Regarding walking onset (*p* = 0.041), the Spanish children started walking earlier than their Ecuadorian peers. On the other hand, the Ecuadorian children showed a lower socio-economic status with respect to the Spanish children (80.7% vs. 30.1%; *p* < 0.001). Regarding education level, the Spanish children’s parents had a higher level of education (61.9%) compared to the parents of the Ecuadorian children (40.8%) (*p* < 0.001). Moreover, 83.4% of the Spanish children’s parents were married, whereas 53.4% of the Ecuadorian children’s parents were married (*p* < 0.001).

Table 1 shows the differences in anthropometric measurements and PF between the Ecuadorian and Spanish children. The Spanish schoolchildren reported better performance in handgrip strength (*p* = 0.021), standing long jump (*p* < 0.001), 25 m running speed (*p* < 0.001), and Léger test (*p* < 0.001), VO_2_ max (*p*= 0.020), as well as a higher rating on Borg’s effort scale (*p* < 0.001) compared to the Ecuadorian children. In addition, the Spanish schoolchildren showed better PF (Z-score) than their Latin American peers (*p* < 0.001).

**Figure 2:**
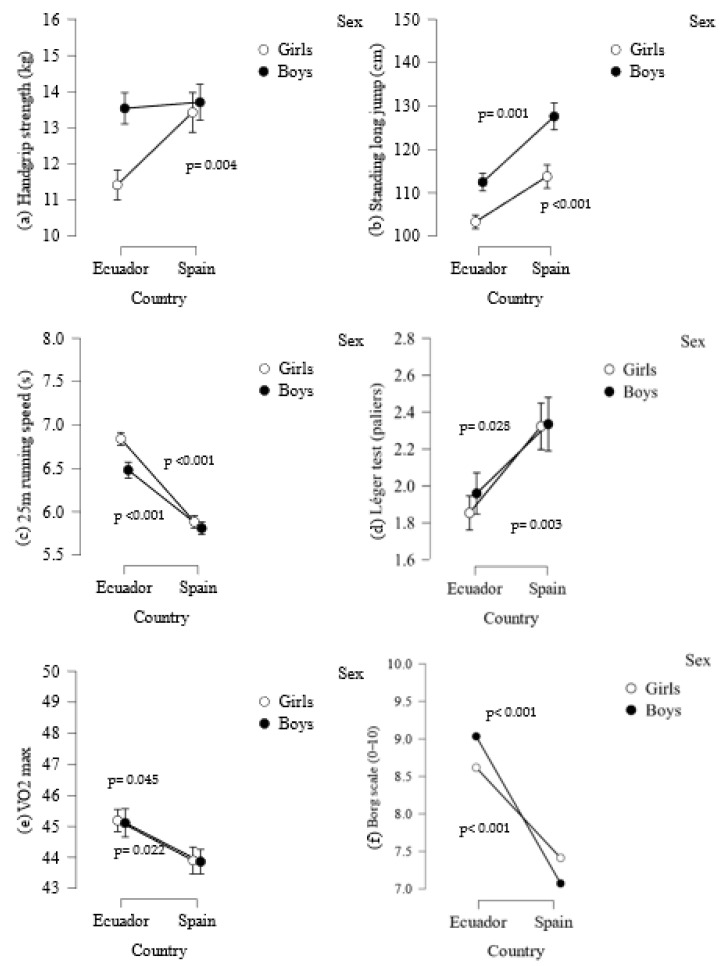
Physical fitness measures in each sex between countries. From a to f, demonstrate the significant differences observed in each physical fitness test between countries by sex, highlighting a higher performance of Spanish children compared to their peers.

Figure 2 compares the results obtained from the preliminary ANOVA for each sex between countries. Figure 2a shows that Spanish girls had better handgrip strength (F [1.170] = 8.670; *p* = 0.004) than the Ecuadorian girls, while no differences were found between the boys. In the standing long jump test, the Spanish girls (F [1.170] = 11.636; *p* < 0.001) and boys (F [1.180] = 16.194; *p* = 0.001) demonstrated greater muscle strength in the lower body with respect to the Ecuadorian children (Figure 2b). Moreover, the Spanish girls and boys (F [1.170] = 87.875, *p* < 0.001; F [1.180] = 34.530, *p* = 0.001, respectively) were faster than their Ecuadorian peers in the 25 m sprint test (Figure 2c). 

Regarding performance in the Léger test, the Spanish girls (F [1.170] = 9.083; *p* = 0.003) and boys (F [1.180] = 4.893; *p* = 0.028) (Figure 2d) showed better performance than the Ecuadorian children. Hence, better VO_2_ max levels were observed in the Spanish girls (F [1.170] = 4.085; *p* = 0.045) and boys (F [1.180] = 5.306; *p* = 0.022) (Figure 2e). Figure 2f highlights the results of Borg’s scale. Both Spanish girls (F [1.170] = 47.455; *p* < 0.001) and boys (F [1.180] = 35.541; *p* < 0.001) showed a higher perception of effort in Léger test performance than the Ecuadorian children.

Table 2 shows the results of the partial correlation analysis (adjusted by sex and age) between breastfeeding period, walking onset, WtHR and anthropometric measurements, and physical performance test. Pearson correlation analysis showed significant correlations between the WtHR and BMI (r = 0.656, *p* < 0.001), 25 m running speed (r = 0.213, *p* < 0.001), and standing long jump (r = −0.261, *p* < 0.001). Moreover, breastfeeding period was associated with 25 m running speed (r= −0.295, *p* < 0.001), walking onset (r= −0.476, *p* < 0.001), Léger test (r = 0.204, *p* < 0.001), and VO_2_ max (r = 0.205, *p* < 0.001), whereas walking onset showed associations with 25 m running speed (r = 0.245, *p* < 0.001), Léger test (r = −0.207, *p* < 0.001), and VO_2_ max (r = −0.201, *p* < 0.001).

In order to further examine the relationship between breastfeeding period and walking onset, regression analysis (method-entering) was performed for physical performance (Table 2). Breastfeeding period and walking onset were considered as predictors. Results indicate that the Ecuadorian and Spanish children showed an association in the standing long jump, 25 m running speed, Leger test (periods), and VO_2_ max with breastfeeding period and walking onset (*p* ranged from <0.001 to 0.028). In addition, the Spanish children showed an association between BMI and breastfeeding period (*p* = 0.043) and between handgrip strength test results and walking onset (*p* = 0.028).

Table 3 shows PF components associated with AO in schoolchildren. The Ecuadorian children showed that 25 m running speed (OR = 2.646, *p* = 0.001), Leger test (OR = 0.674, *p* = 0.018), and VO_2_ max (OR = 0.846, *p* = 0.021) were protective factors of AO. Moreover, handgrip strength (OR = 1.084, *p* = 0.039) and PF Z score (OR = 0.344, *p* = 0.031) were protective factors of AO in the Spanish children. In both the Ecuadorian and Spanish children, standing long jump (OR = 0.972, *p* = 0.021 and OR = 0.985, *p* = 0.047, respectively) and Léger test (OR = 0.674, *p* = 0.018 and OR = 0.688, *p* = 0.031, respectively) were protective components of AO.

## 4. Discussion

The aim of this study was to compare levels of PF between Ecuador and Spain and identify whether breastfeeding period, walking onset, and AO are determinants of physical performance in schoolchildren from Ecuador and Spain. To our knowledge, this study is among the few studies that address the association of these factors with PF performance in schoolchildren. These results corroborate our first hypothesis, as differences were observed in PF for strength, speed, and endurance according to geographical location, with Spanish children showing better physical performance than Ecuadorian children. 

In the same line, a study carried out by Luz et al. [43] among 508 Portuguese and 710 American children aged 6–13 years, which assessed PF using some of the same physical tests as in the present study, concluded that, regardless of sex, Portuguese children showed better PF and motor development than American children, while American children were better in manual coordination tests. The authors suggest that these differences may be due to the Portuguese curriculum guidelines that place special emphasis on the development of physical and motor activities in early childhood, whereas the US curriculum tends to focus on free play, with little focus on gross motor development guidelines in preschools. 

A cross-national study that included 347,935 students aged 15–16 from the 52 PISA countries analyzed the association between hours allocated to the physical education curriculum, as well as wealth and income inequality between countries, and concluded that Hungary and Poland showed higher levels of physical activity than the Eastern Mediterranean region (Spain) and were higher than the participating countries in the Americas. The authors point out that these discrepancies could be due to the influence of different determinants, such as the individual development, social development, and environmental and public policies in each country [44].

These differences may be explained by the development and influence of cultural differences, as well as the impact of physical activity levels and physical education curriculum between countries [45]. Moreover, adherence to the Mediterranean diet in Spanish children is predictive for health-related quality of life and high fitness status [46]. On the other hand, a meta-analysis conducted by Godoy-Cumillaf et al. [47] highlights that there is a lack of scientific evidence on PF in Latin American children and adolescents, as well as a lack of updated normative values for the American population. 

Another possible explanation for these findings could be attributed to the economic gap, as it can exert a substantial influence on access to resources such as healthy food, sports facilities, and medical care, which in turn can influence children’s health and physical development from an early age [48]. Furthermore, socioeconomic disparities are also associated with an increased risk of abdominal obesity and related health problems in childhood [49].

Regarding our second hypothesis, both the breastfeeding period and walking onset were determinants of physical performance in Ecuadorian and Spanish children. Vafa et al. [16] who showed in a retrospective cohort study among 246 schoolchildren (categorized in three groups: over 6 months of breastfeeding, less than 6 months of breastfeeding, and formula) that cardiorespiratory fitness was positively associated with over 6 months of breastfeeding (VO_2_ max was significantly higher in the >6 months group). These findings further support the idea that exclusive breastfeeding for more than 6 months has been shown to have a beneficial effect on PF performance in childhood. Additionally, healthy nutrition (breastfeeding) may serve as a predictor for the physical health of children and adolescents [16]. Labayen et al. [18] concluded that after studying the effect of breastfeeding duration and cardiorespiratory fitness among 1025 schoolchildren, the longer period of breastfeeding was positively associated with cardiorespiratory fitness. A possible explanation might be that exclusive breastfeeding can have advantages due to special nutrients, such as trophic substances, hormones, and long-chain polyunsaturated fatty acids, which are not present in formula milk [17]. Furthermore, in a sample of 2853 European children from the IDEFICS research aged 6–11 years using the same tests as in the present study, Zaqout et al. [50] showed a positive association between breastfeeding and lower muscular explosive strength. The authors concluded that exclusive breastfeeding between 1 and 3 months showed an influence on lower limb explosive strength, while exclusive breastfeeding between 4 and 6 months showed benefits in flexibility/balance. Vafa et al. [16] concluded that the period of exclusive breastfeeding for more than 6 months showed positive effects on cardiorespiratory fitness, indicating that early nutrition may be a predictor of PF throughout adolescence.

Moreover, Artero et al. [51] found that time of exclusive breastfeeding or mixed (exclusive breastfeeding + formula feeding) was positively associated with lower body explosive strength, independent of morphological or other possible confounding factors. found that time of exclusive breastfeeding or mixed feeding (exclusive breastfeeding + formula feeding) was positively associated with lower body explosive strength, independent of morphological or other possible confounding factors. These findings imply that breastfeeding may have a role in influencing lower body explosive strength during adolescence. In contrast, Lawlor et al. [15] showed a positive association between anthropometric factors and cardiorespiratory fitness; however, they did not find an association between breastfeeding and cardiorespiratory fitness. On the other hand, certainly, the educational and employment status of mothers significantly influences the duration of breastfeeding. Previous research shows that mothers with higher education and stable employment breastfeed for longer periods [52,53]. These mothers tend to have better access to resources such as maternity leave and breastfeeding support, which facilitates longer durations of breastfeeding [53]. Overall, addressing barriers related to education and employment is crucial for promoting breastfeeding in diverse populations [54].

Regarding walking onset in children and PF, the current study makes a significant contribution to early motor development by demonstrating the relationship between walking onset and PF performance. These findings identify potential links between gait initiation and aspects of PF, and the study lays the foundation for future investigations into the factors influencing motor milestones, talent identification, and their implications for overall health. These findings further support the idea that late onset of walking is correlated with poorer motor skills, including fine motor skills and static and dynamic balance, as well as poorer cognitive skills, such as selective attention and visual perception, at the end of pre-school age [9]. Furthermore, Liu et al. [7] highlighted that gait patterns showed associations with the maturation of the neuro- and musculoskeletal systems in children.

Another important finding was that PF was a common protective factor between Ecuadoran and Spanish schoolchildren against AO. In this sense, Caamaño-Navarrete et al. [55] found that AO and excessive weight were linked with an unhealthy lifestyle in Latin American schoolchildren. The findings in the current study are consistent with those of Latorre-Román et al. [23], who showed that Chilean children exhibited a higher prevalence of AO, with significant differences compared to Colombian and Spanish girls. AO is linked to an increased risk of cardiovascular disease and type 2 diabetes. In this sense, higher AO, associated with a hypercaloric diet and low levels of physical activity, is significantly related to poor PF during childhood [56]. In general, the present findings contribute to an expanding body of literature on children and adolescents regarding the association between PF and AO. Thus, improving PF through physical activity promotion is a key goal in lifestyle interventions aimed at improving children’s health [57].

### 4.1. Strength and Limitations

One of the strengths of this study was that it analyzed in a single study the associations between PF and breastfeeding period, walking onset, and AO in schoolchildren. However, further large studies with long-term follow-up are needed to replicate these findings. However, there are some limitations that should be mentioned. The cross-sectional design of this study restricts the scope of the conclusions. Another limitation is the self-supplied information in the ad hoc questionnaire by parents, as there may be underreporting or overreporting. Acknowledging these limitations is crucial for interpreting the study’s results accurately and for guiding future research endeavors in understanding the complex interplay between early-life factors and children’s PF. 

### 4.2. Practical Application

The findings of this study provide tangible guidance for early identification of health concerns in preschool children and emphasize the need for comprehensive interventions, including nutritional education and increased physical activity, to foster a healthier environment within the school setting. Therefore, these results may support the idea of including physical activity guidelines in schools from an early stage, as well as enhancing the large-scale findings on the importance of post-natal factors in children’s motor development as a long-term health and well-being policy.

## 5. Conclusion

The results of this study underscore the influence of early-life factors, such as breastfeeding duration and walking onset, as well as the impact of AO on physical performance in schoolchildren. Additionally, the observed differences in PF between Ecuadorian and Spanish children highlight potential variations in lifestyle, environment, or genetic factors between the two populations. In turn, these differences highlight public policy approaches to child well-being across continents. Hence, this study contributes valuable insights into the multifaceted determinants of PF in schoolchildren, emphasizing the importance of considering both biological and environmental factors in promoting and understanding children’s health.

## Figures and Tables

**Table 1:** Anthropometric measures and physical performance in schoolchildren across countries.

	ALL		Ecuador		Spain			
Variables	(n = 352)		(n = 176)		(n = 176)			
	Mean (SD)		Mean (SD)		Mean (SD)		*p*-Value	Cohen’s d
Age (years)	8.54	(1.79)	8.47	(1.74)	8.61	(1.85)	0.460	0.078
Weight (kg)	34.99	(11.06)	34.64	(10.49)	35.35	(11.64)	0.549	0.064
Height (m)	1.35	(0.12)	1.33	(0.11)	1.36	(0.13)	0.046	0.249
BMI (kg/$m^{2}$)	18.73	(4.19)	19.04	(4.35)	18.42	(4.02)	0.164	0.148
Waist circumference (cm)	63.66	(10.56)	64.55	(11.04)	62.77	(10.0)	0.115	0.169
WtHR (WC/height)	0.47	(0.07)	0.46	(0.09)	0.47	(0.06)	0.754	0.131
Underweight	4	1.1%	3	1.7%	1	0.6%	0.696	
Healthy weight	213	60.5%	105	59.7%	108	61.4%		
Overweight	96	27.3%	50	28.4%	46	26.1%		
Obese	39	11.1%	18	10.2%	21	11.9%		
Handgrip strength (kg)	13.02	(4.52)	12.46	(4.09)	13.57	(4.87)	0.021	0.247
Standing long jump (cm)	114.49	(24.46)	107.83	(17.57)	121.15	(28.32)	<0.001	0.566
25 m running speed (s)	6.25	(0.83)	6.66	(0.79)	5.84	(0.65)	<0.001	1.136
Léger test (periods)	2.11	(1.15)	1.89	(1.05)	2.33	(1.21)	<0.001	0.389
${VO}_{2}$ max. (mL·${kg}^{-1}$ 1·${min}^{-1}$)	44.51	(3.88)	43.88	(3.85)	45.15	(3.83)	0.002	0.331
Physical fitness Z score	0.04	(0.99)	−0.25	(0.73)	0.33	(1.13)	<0.001	0.611
Borg scale	8.02	(1.82)	7.23	(2.03)	8.82	(1.13)	<0.001	0.970

WtHR = waist-to-height ratio; WC = waist circumference; BMI: body mass index.

**Table 2:** Standardized beta coefficients from linear regression models adjusted on the breastfeeding period and walking onset with physical performance.

Variables			BMI		Handgrip Strength		Standing Long Jump		25 m Running Speed		Léger Test		${VO}_{2}$ Max	
			Adjus Ted	Raw	Adjus Ted	Raw	Adjus Ted	Raw	Adjus Ted	Raw	Adjus Ted	Raw	Adjus Ted	Raw
Breastfeeding period	Ecuador	β	−0.139	−0.131	0.008	0.135	0.238	0.310	−0.316	−0.409	0.209	0.246	0.150	0.008
		*p*-value	0.071	0.085	0.891	0.075	0.001	<0.001	<0.001	<0.001	0.005	0.001	0.006	0.919
	Spain	β	−0.168	−0.153	0.043	0.015	0.143	0.103	−0.206	−0.188	0.060	0.051	0.042	0.075
		*p*-value	0.028	0.043	0.444	0.847	0.021	0.178	0.004	0.013	0.792	0.505	0.377	0.327
Walking onset	Ecuador	β	0.061	0.065	0.058	0.067	−0.201	−0.190	0.232	0.233	−0.182	−0.174	−0.128	−0.124
		*p*-value	0.420	0.390	0.328	0.377	0.003	0.012	<0.001	0.003	0.013	0.021	0.017	0.101
	Spain	β	0.168	0.162	−0.103	−0.167	−0.120	−0.199	0.252	0.284	−0.207	−0.222	−0.128	−0.625
		*p*-value	0.030	0.032	0.082	0.028	0.056	0.009	0.001	<0.001	0.006	<0.001	0.008	0.533

**Table 3:** Fitness measures associated with AO index according to country.

	Abdominal Obesity			
Variables	Ecuador		Spain	
	OR (CI95%)	*p*-Value	OR (CI95%)	*p*-Value
Handgrip strength (kg)	0.979 (0.882–1.086)	0.683	1.084 (1.004–1.004)	0.039
Standing long jump (cm)	0.972 (0.950–0.996)	0.021	0.985 (0.970–1.000)	0.047
25 m running speed (s)	2.646 (1.515–4.622)	0.001	0.828 (0.509–1.347)	0.448
Léger test (periods)	0.674 (0.487–0.934)	0.018	0.688 (0.490–0.966)	0.031
${VO}_{2}$ max. (mL·${kg}^{-1}$ 1·${min}^{-1}$)	0.846 (0.7363–0.972)	0.021	1.024 (0.936–1.120)	0.611
Physical fitness Z score	0.473 (0.197–1.132)	0.093	0.344 (0.031–0.906)	0.031

Note: Data displayed represent OR (95% CI), adjusted for age and sex.

## Data Availability

Data supporting this research can be requested from the corresponding author.
